# A novel minimally invasive OFM technique with orthotopic transplantation of hUC-MSCs and in vivo monitoring of liver metabolic microenvironment in liver fibrosis treatment

**DOI:** 10.1186/s13287-021-02599-w

**Published:** 2021-10-09

**Authors:** Hui Yang, Yuanyuan Xie, Tuo Li, Shuo Liu, Sheng Zeng, Bin Wang

**Affiliations:** 1 Center for Clinic Stem Cell Research, The Affiliated Drum Tower Hospital of Nanjing University Medical School, 321 Zhongshan Road, Nanjing, 210008 Jiangsu China; 2 Department of Nuclear Medicine, Peking Union Medical College Hospital, Beijing, 100730 China

**Keywords:** Umbilical cord mesenchymal stem cells, Liver fibrosis, Orthotopic transplantation, Open-flow microperfusion, Microenvironment, Metabolomics

## Abstract

**Background:**

Mesenchymal stromal cells (MSCs) transplantation showed promising therapeutic results in liver fibrosis. However, efficient cell delivery method is urgently needed and the therapeutic mechanism remains unclear. This study focused on developing a minimally invasive open-flow microperfusion (OFM) technique, which combined orthotopic transplantation of human umbilical cord-derived (hUC)-MSCs to liver and in vivo monitoring of liver microenvironment in mice with CCl_4_-induced liver fibrosis.

**Methods:**

The therapeutic potential of OFM route was evaluated by comparing OFM with intravenous (IV) injection route in terms of hUC-MSCs engraftment at the fibrosis liver, liver histopathological features, liver function and fibrotic markers expression after hUC-MSCs administration. OFM was also applied to sample liver interstitial fluid in vivo, and subsequent metabolomic analysis was performed to investigate metabolic changes in liver microenvironment.

**Results:**

Compared with IV route, OFM route caused more hUC-MSCs accumulation in the liver and was more effective in improving the remodeling of liver structure and reducing collagen deposition in fibrotic liver. OFM transplantation of hUC-MSCs reduced blood ALT, AST, ALP and TBIL levels and increased ALB levels, to a greater extent than IV route. And OFM route appeared to have a more pronounced effect on ameliorating the CCl_4_-induced up-regulation of the fibrotic markers, such as α-SMA, collagen I and TGF-β. In vivo monitoring of liver microenvironment demonstrated the metabolic perturbations induced by pathological condition and treatment intervention. Two metabolites and eight metabolic pathways, which were most likely to be associated with the liver fibrosis progression, were regulated by hUC-MSCs administration.

**Conclusion:**

The results demonstrated that the novel OFM technique would be useful for hUC-MSCs transplantation in liver fibrosis treatment and for monitoring of the liver metabolic microenvironment to explore the underlying therapeutic mechanisms.

**Supplementary Information:**

The online version contains supplementary material available at 10.1186/s13287-021-02599-w.

## Background

Liver fibrosis remains a serious health problem which affects a significant number of people all over the world [1]. The onset of liver fibrosis is typically attributed to a variety of pathogenic factors, including viral hepatitis, alcohol abuse, drug toxicity, autoimmunity, and so on [2]. The chronic injuries lead to a wound-healing response, which is characterized by a switch of hepatic stellate cells (HSCs) from quiescent to an activated myofibroblast-like phenotype [3]. As the central mediators of fibrogenesis, activated HSCs release profibrogenic factors and cytokines, such as transforming growth factor beta (TGF-β) and alpha-smooth muscle actin (α-SMA) [4–6]. Moreover, liver fibrogenesis accompanies excessive production of extracellular matrix (ECM) to reconstruct the intrahepatic structure, which would further contribute to liver cirrhosis and hepatocellular carcinoma (HCC) [7]. The reversal of liver fibrosis is crucially important for reducing the mortality relevant to liver cirrhosis and HCC. Thus far, there currently is no effective therapy for treatment of liver fibrosis. Although liver transplantation seems to be a preferred strategy, there remain a number of challenges in this method, including shortage of donor organs, immune rejection response and surgery complications [8, 9]. Hence, it is urgent to search for an alternative treatment strategy.

Currently, mesenchymal stromal cell (MSC) therapy is regarded as a promising strategy for liver fibrosis treatment [10] because MSCs possess various advantageous characteristics, such as continuous self-renewal, strong proliferative ability, immunomodulatory activities, and multidifferentiation potential [11, 12]. The human umbilical cord-derived (hUC)-MSCs exhibit not only the general characters of MSCs but also relatively easy accessibility, abundant source, no substantial ethical issues and more stable biological properties, making the use of hUC-MSCs a superior choice for liver fibrosis treatment [13, 14]. A series of studies have demonstrated the efficacy of hUC-MSCs therapy in liver fibrosis [15].

Up to now, the translation of numerous MSC therapies has not fully realized their clinical application potential. One critical but often overlooked challenge has been the administration route of MSCs; indeed, many studies have revealed when the same MSC therapy is introduced through different routes of administration, the engraftment and retention of MSCs within the liver are different, which results in different therapeutic outcomes [16, 17]. Compared with the common method of clinical systemic administration route: intravenous (IV) injection, which results in “lung entrapment” [18, 19], directly delivering cells to the target tissue such as intraportal and intrahepatic injection is increasingly considered to be able to increase the therapeutic effects [12]. Hence, the development of an orthotopic injection route is urgently required to improve MSC delivery to preclinical liver fibrosis models.

The mechanisms of MSC-based liver fibrosis therapy have not been fully delineated [20], which is another reason why the clinical application of MSC-based therapy has not been fully exploited. Growing researches suggest that a more comprehensive assessment of the change of the tissue microenvironment components during the progression of the disease and healing process gives new clues for the mechanism studies [21, 22]. However, there is no report focusing on monitoring liver microenvironment during liver fibrosis progression and MSCs treatment process. To fill the research gap, a minimally-invasive in vivo sampling technique that allows continuous sampling of liver microenvironment components is required.

In this study, a novel minimally-invasive open-flow microperfusion (OFM) route was developed to improve hUC-MSCs delivery efficiency to the liver for liver fibrosis treatment. MSCs entrapment in liver and therapeutic effects of OFM route were compared with IV route. In addition, OFM was simultaneously employed to sample the liver interstitial fluid in vivo during fibrosis progression and healing process of liver fibrosis, and subsequently combined with metabolomic analysis. This allowed for the investigation of the potential therapeutic mechanisms.

## Methods

### Isolation and culture of hUC-MSCs

With informed consent, the hUC-MSCs were isolated from fresh umbilical cord of a full-term delivery donor. The entire procedure was approved by the Medical Ethics Committee of The Affiliated Drum Tower Hospital of Nanjing University Medical School. The umbilical cord was washed with sterile PBS and was cut into 3–5 mm long pieces, suspended in Dulbecco's modified Eagle's medium–low glucose (DMEM-LG, Gibco, USA) supplemented with 10% FBS (Gibco, USA) and 1% penicillin/streptomycin (Gibco, USA). Then the isolated cells were cultured in an atmosphere of 5% CO_2_ at 37 °C and the medium was replaced every 3–4 days until well-developed colonies of fibroblast-like cells appeared. The cells were then digested with 0.25% trypsin and seeded in new culture bottles for further expansion. The cells of fourth generation were harvested as purified hUC-MSCs and taken for further studies. The adhesiveness properties were identified and the morphological characteristics of hUC-MSCs were detected by an inverted microscope (Olympus, Japan). Flow cytometry (BD facsaria^tm^, USA) was employed for hUC-MSCs characterization according to published protocols [23], data analysis was performed using FACS software.

### Liver fibrosis model

C57BL/6 mice (7–8 weeks old, weighing 19–23 g) were purchased from Nanjing Medical University and housed in a temperature/humidity-controlled environment with light illumination cycles of 12 h/day and had free access to diet and water. The animal experiments were approved by the Institutional Animal Care and Use Committee of Nanjing University. To induce liver fibrosis, CCl_4_ was dissolved in olive oil at the volume ratio of 1:3. Mice were intraperitoneally injected with 150 μL of CCl_4_ solution twice a week for eight weeks. The normal group received 150 μL olive oil twice a week for eight weeks.

### Fabrication of OFM probe

A polyimide capillary (O.D. 0.38 mm, I.D. 0.28 mm) was used for the fabrication of the OFM probe. The capillary was perforated with 60 holes at intervals of about 50 μm on a laser ablation platform (NWR-213 system, Electro Scientific Industries, USA). The diameter of the hole was 100 μm.

### Transplantation of hUC-MSCs

After induction of liver fibrosis, CCl_4_-exposed mice were randomly divided into a model group, a hUC-MSCs OFM-treated group, and a hUC-MSCs IV-treated group. In hUC-MSCs OFM-treated group, the mouse was anesthetized and the abdominal cavity of the mouse was opened. OFM probe with 60 holes (100 μm diameter) was implanted into the left lobe of liver under the traction of catheter. One end of the probe was plugged with an empty syringe, another end of the probe was connected to a push pump and 1 × 10^6^ hUC-MSCs in 300 μL PBS were delivered at the flow rate of 25 μL/min. In IV group, same amount of hUC-MSCs was injected to the caudal vein of the mouse. In model group, mice were administrated with 300 μL PBS at a flow rate of 25 μL/min via OFM route.

### Cell labeling and homing experiments in vivo

hUC-MSCs were labeled by dyechloromethylbenzamido-1,1’-dioctadecyl-3,3,3’,3’-tetramethyl indocarbocyanine perchlorate (CM-Dil, Sigma-Aldrich, USA) in accordance with the manufacturer’s guidance. After digestion and centrifugation, 6 × 10^6^ hUC-MSCs were resuspended in 400 μL CM-Dil solution with concentration of 20 μg/mL. The mixed suspension was incubated at 37 °C for 15 min, then at 4 °C for 15 min. Then, the labeled-cells were washed with PBS 3 times to eliminate free CM-Dil residual. Finally, the labeled hUC-MSCs suspension was prepared with a concentration of 3.3 × 10^6^ cells/mL. To evaluate the distribution of hUC-MSCs in liver fibrosis model at day 3, 7, 14 and 21 post-transplantation, the same amount of CM-Dil-labeled hUC-MSCs were administrated using transplantation method of OFM or IV as described above. Mice were sacrificed 3, 7, 14 or 21 days after cell transplantation. Livers, lungs, spleens, hearts and kidneys were collected and fixed in 4% (v/v) paraformaldehyde (PFA) overnights. Then the tissues were transferred to 20% (w/v) sugar for 12 h and 30% (w/v) sugar overnight for dehydration. After dehydration, the tissues were embedded in chilled OCT and frozen at − 80 °C. Cryosections of liver, lung, spleen, heart and kidney samples were prepared with a thickness of 12-μm (three slides each sample) and incubated with DAPI dye (Sigma-Aldrich, USA) in the dark. The distribution of CM-Dil-labeled cells in different tissues was observed by confocal microscope (Leica Microsystems, Germany).

### Serum biochemical indicators

Serum samples were collected and serum alanine aminotransferase (ALT), aspartate aminotransferase (AST), alkaline phosphatase (ALP), total bilirubin (TBIL), and albumin (ALB) levels were measured on day 21 post-MSCs transplantation by a chemistry analyzer (VITROS 5600, USA).

### Histopathological and immunohistochemical evaluation

Liver tissues were removed from mice instantly after euthanization on day 21 post-MSCs transplantation and divided into sections. One section was washed with PBS, fixed in 4% PFA and embedded in paraffin. Then the liver samples were cut into 4 μm-thickness slices. To illustrate the histological details, hematoxylin&eosin (H&E) staining was performed. To evaluatethe collagen deposition, masson's trichrome and sirius-red was performed. Immunohistochemistry (IHC) staining was also performed with antibodies against α-SMA (abcam, UK), collagen I (abcam, UK) or TGF-β (abcam, UK). The staining results were detected with an optical microscope (Leica, Germany).

### Evaluation of fibrotic markers

Total RNA was harvested from another unmanipulated liver tissue using TRIzol® reagent (Invitrogen, USA) and was reverse-transcribed into cDNA with HiScript®III RT SuperMix for qPCR (Vazyme Biotech Co., Ltd, China) using 10 μg RNA. ChamQ Universal SYBR qPCR Master Mix (Vazyme Biotech Co., Ltd, China) was used for template amplification with a primer for each of the transcripts examined. PCR reagents were assessed by a three color real-time PCR machine (Applied Biosystems,Carlsbad, CA). All reactions repeated three times. Relative quantification of gene expression was performed through normalizing to the expression of β-actin as an internal control. The mRNA expression levels of α-SMA, collagen I, and TGF-β in normal group, model group, hUC-MSCs OFM-treated group, and hUC-MSCs IV-treated group were compared. The primer sequences including α-SMA, collagen I, TGF-β, and β-actin were listed in Table 1.

**Table 1:** Primer sequences

Gene name	Primer sequences (5′-3′)
α-SMA	GAACACGGCATCATCACCAAC CTCCAGAGTCCAGCACAATACC
Collagen I	GCTCCTCTTAGGGGCCACT
	CCACGTCTCACCATTGGGG
TGF-β	GCCCTGGATACCAACTATTGC
	GCAGGAGCGCACAATCATGTT
β-Actin	GGCTGTATTCCCCTCCATCG CCAGTTGGTAACAATGCCATGT

### OFM sampling

OFM sampling of liver interstitial fluid was performed in normal group, model group and hUC-MSCs OFM-treated group. The operation of OFM sampling was similar to OFM transplantation of hUC-MSCs described above with a little modification. In brief, one end of the OFM probe was connected to a push pump (Cole-Parmer, USA) and another end of the OFM probe was connected to a pull pump (Harvard Apparatus, USA) after implantation into the liver. The perfusion fluid was normal saline. The flow rate of the push pump and the pull pump was 2 μL/min. The probe was perfused for 30 min for equilibration, then 80 μL dialysate sample was obtained from each mouse. The dialysates were stored at -80 °C until the time of analysis.

### UPLC-ESI HR MS/MS

The liver dialysates obtained by OFM sampling were analyzed using ultra performance liquid chromatography-electrospray high resolution MS/MS (UPLC-ESI HR MS/MS). In brief, 80 μL dialysate sample was dried with a vacuum dryer, and then re-dissolved in 50 μL of ethanol containing 0.25 μg/mL isoprenaline (Sigma-Aldrich, USA) as internal standard (IS). The mixture was centrifuged for 15 min at a rate of 10,000*g* at 4 °C. The supernatant was collected for UPLC-ESI HR MS/MS analysis. Samples were loaded into the UPLC system (Ultimate 3000, Thermo Fisher Scientific, USA) equipped with a BEH amide column (1.7 μm, 2.1 mm ID × 20 mm, Waters, USA). The mobile phase A was water containing 20 mM ammonium acetate and the mobile phase B was acetonitrile containing 0.1% formic acid. The LC separations were 30 min per sample with a flow rate at 0.3 mL/min using LC gradient reported in the previous literature [24]. The metabolite profile was acquired using Orbitrap Fusion Lumos MS (Thermo Fisher Scientific, USA) with positive-ion mode. Major operating parameters were as follows: electrospray voltage + 3000 V, *m/z* range 150–1000, ion transfer tube temperature 325 °C, vaporizer temperature 275 °C, sheath gas flow 30 Arb, auxiliary gas flow 10 Arb. Metabolite fragments were obtained under high energy collisional dissociation (HCD) mode with a collision energy at 20 eV.

### Metabolite identification

The high resolution MS spectra and MS/MS spectra were loaded into Compound Discovery (Thermo Fisher Scientific, USA). Database search was performed after peak alignment and peak area integration. The putative annotation of the metabolites was achieved by precisely matching mass with mzCloud, ChemSpider and MassList databases. Further structural confirmation was performed by MS/MS fragmentations obtained in a data dependent acquisition mode. The concentration data of the hUC-MSCs OFM-treated group, the model group and the normal group were acquired by comparing the peak area of each metabolite with that of IS. Principal component analysis (PCA) was performed using SIMCA-P 14.1 (Umetrics AB, Sweden) with the concentration data. Volcano plot and heat map were obtained using Graphpad Prism5.0 (GraphPad Software, USA) with the concentration data. Metabolic pathway analysis was performed on MetaboAnalyt website (https://www.metaboanalyst.ca).

### Statistical analysis

The data generated by the experiments described above were presented as mean ± standard error of the mean (SEM). Graphpad Prism 5.0 was used to generate graphs. Statistical significance of differences between groups was evaluated using a standard one-way analysis of variance (ANOVA) or non-parametric test. A *p* value of less than 0.05 was considered statistically significant.

## Results

### Characterization of isolated hUC-MSCs

The hUC-MSCs exhibited typical fibroblastic-like morphology (Additional file 1: Fig. 1A). The immune phenotypes of hUC-MSCs were characterized by flow cytometry. As shown in Additional file 1: Fig. 1B, the cells positively expressed CD73, CD90, CD105, and CD29, which are MSC-specific surface antigens. However, they lacked other markers, such as CD34, CD45, CD117, and human leukocyte antigen-DR (HLA-DR).

**Fig. 1:**
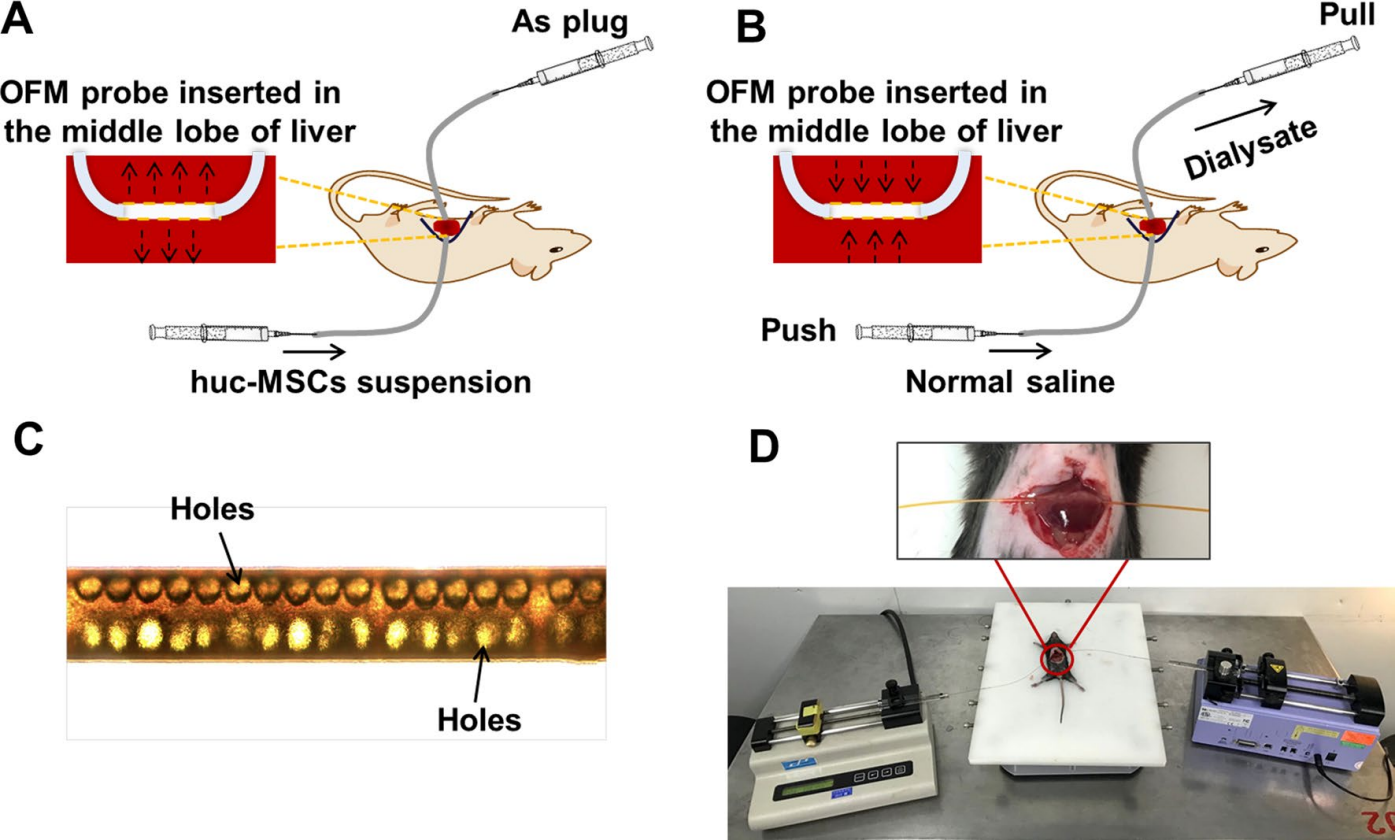
The schematic diagram of OFM. **A** Scheme of hUC MSCs transplantation via OFM route. OFM probe was implanted into the liver. One end of the probe was plugged with an empty syringe, another end of the probe was connected to a push pump and hUC-MSCs suspension was delivered at constant flow rate. **B** Scheme of OFM sampling of liver interstitial fluid in vivo and in situ. One end of the OFM probe after implantation into the liver was connected to a push pump and another end of the OFM probe was connected to a pull pump. The perfusion fluid was normal saline. The flow rate of the push pump and the pull pump was equal. **C** Microscope image of OFM probe. **D** Photograph of OFM probe inserted in liver of anesthetized mice

### OFM route allowed a greater number of transplanted cells to seed into the fibrotic liver

**Fig. 2:**
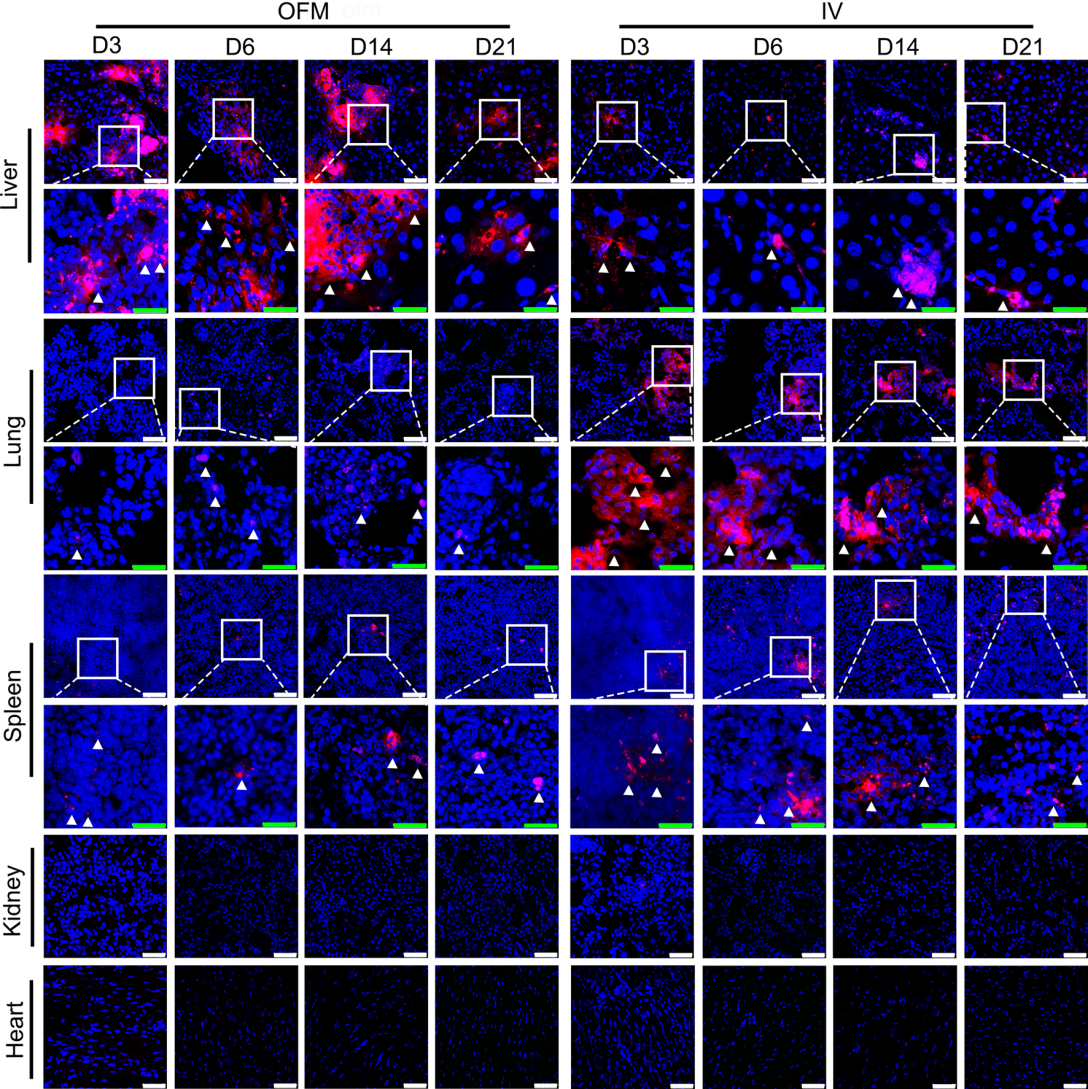
CM-Dil-labeled hUC-MSCs (red) distribution analysis on liver, lung, spleen, kidney and heart at day 3, 6, 14 and 21 post transplantation via OFM and IV route, respectively. Scale bar (white) = 50 μm, scale bar (green) = 25 μm

OFM is a minimally invasive, universal and continuous in vivo sampling technique, which is widely used in sampling interstitial fluid components via macroscopic holes without nominal cut-off value [25]. In this study, to our knowledge, OFM was firstly employed for orthotopic transplantation of hUC-MSCs into the fibrotic liver. As shown in Fig. 1A, D, OFM probe was inserted in the liver and perfused with hUC-MSCs suspension at a constant speed. hUC-MSCs were delivered into the liver through macroscopic holes on the OFM probe (Fig. 1C). The number of transplanted hUC-MSCs that can seed at the injury site is one of the essential prerequisite for successful cell therapy. In order to investigate the homing ability of hUC-MSCs transplanted by OFM route, fluorescent CM-Dil was introduced to label hUC-MSCs. CM-Dil labeling had no distinct effect on cell morphology (Additional file 1: Fig. 1C). CM-Dil-labeled hUC-MSCs were infused into mice with liver fibrosis by OFM and IV route. As shown in Fig. 2, in the OFM group, most of hUC-MSCs were trapped in the liver, the fluorescence signal intensity did not change significantly from day 3 to day 14 after infusion and on day 21, the fluorescence signals decreased obviously. In addition, red fluorescence was weak in lung and spleen though the signal intensity increased slowly as time progressed. However, in the IV group, hUC-MSCs accumulated mainly in the lung while the cell concentration was much lower in the liver. The IV group showed higher hUC-MSCs existence in the spleen than OFM group. There was no CM-Dil staining within the kidney and heart in both OFM and IV group, which indicated hUC-MSCs could not migrate to the kidney and heart.

### OFM made the cell therapy more effective for liver fibrosis

**Fig. 3:**
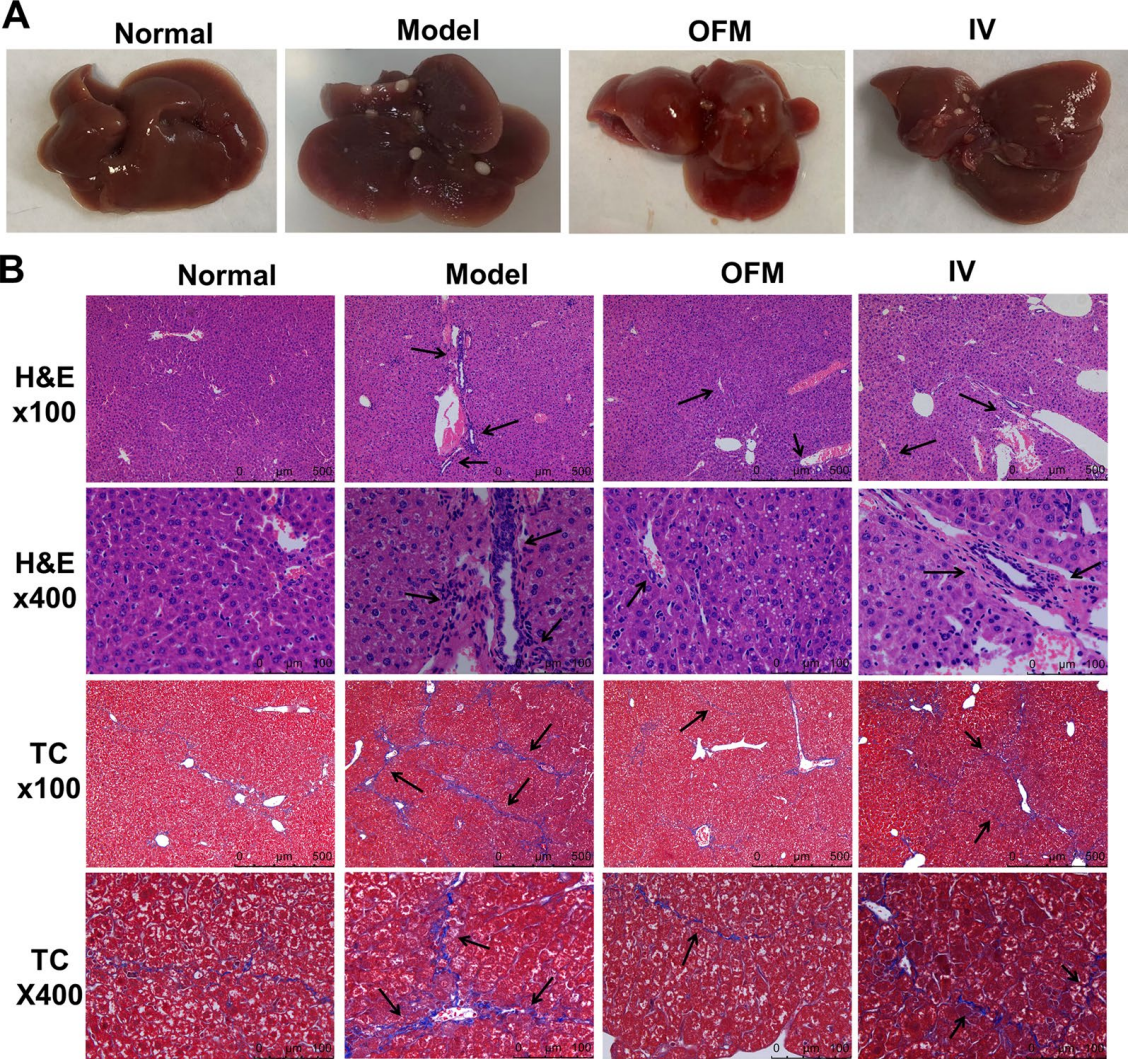
The therapeutic effects on liver fibrosis after hUC-MSCs transplantion via IV or OFM route. **A** Macroscopic observation of the liver condition. **B** Histopathological improvement of liver tissues at day 21 after hUC-MSCs transplantion was evaluated by H&E staining (magnification 100x, 400x, the arrows indicate inflammatory cell infiltration) and masson's trichrome staining (magnification 100x, 400x, the arrows indicate collagen deposition)

Since the OFM route allowed more cells to reside in the fibrotic liver, the effect of OFM route in the treatment of liver fibrosis was further explored and the difference in therapeutic efficacy between OFM and IV routes was compared. Mice were euthanized and livers were collected. The macroscopic condition of the livers was evaluated firstly. As shown in Fig. 3A, the livers obtained from the model group were dark red and characterized by a rough and fractured surface with raised speckle and blunt edge, while livers of the normal group were bright-red and distinguished by a soft and smooth surface with sharp edges. Both OFM and IV routes improved the overall appearance of livers as compared to the model group. In the OFM group, the number of raised speckle was less than that in the IV group.

H&E staining results (Fig. 3B) showed that the structure of liver lobules in normal group was regular, and there was no portal inflammation. In model group, the structure of liver lobules was destroyed, the arrangement of liver cell cords was disordered, the liver cells were denatured and partially necrotic, accompanied by obvious inflammatory cell infiltration in the portal area and around the central vein. Both OFM and IV transplantation dramatically reduced portal inflammation and improved the lobular structural pattern. In addition, from the H&E staining results, the degree of liver injury in OFM group was less than that in IV group.

Masson's trichrome and sirius-red staining results (Fig. 3B) showed that small amounts of collagen fibers were distributed in portal area in normal group, while fibrous septa was detected in model group. OFM and IV transplantation of hUC-MSCs could reduce fibrous expansion around the portal area in different degrees. In comparison with IV group, masson's trichrome staining of livers from OFM group appeared more similar to that of normal group.

**Fig. 4:**
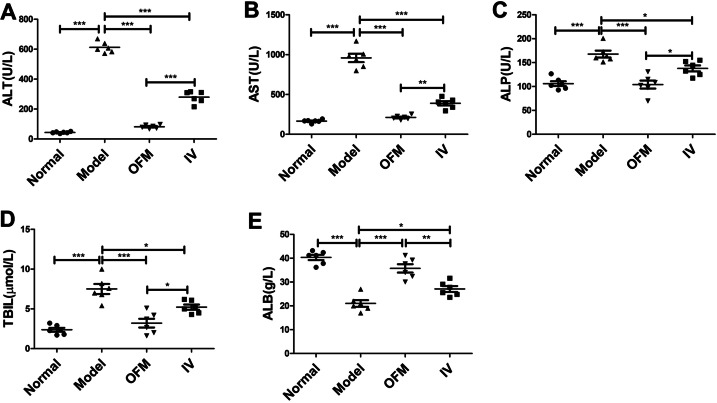
The recovery of liver function of mice with liver fibrosis after hUC-MSCs transplantion was assessed by measuring the levels of serum biochemical indicators. **A–E** The serum ALT, AST, ALP, TBIL, and ALB levels were assayed in four groups. Data were represented as mean ± SEM (*p < 0.05, **p < 0.01, ***p < 0.001)

Liver function was evaluated through measurements of serum biomarkers, including serum ALT, AST, ALP, TBIL, and ALB. As shown in Fig. 4, after 8 weeks of CCl_4_ administration, remarkable increase of serum ALT, AST, ALP, and TBIL was detected in model group, while ALB serum levels were noticeably decreased in the model group. The hUC-MSCs transplantation via OFM or IV route significantly reduced ALT, AST, ALP, and TBIL levels and increased ALB levels in comparison with model group. Serum ALT, AST, ALP, and TBIL levels in OFM group were noticeably lower than those in IV group, and ALB levels in OFM group were significantly higher than those in IV group. It was worth noting that there was no significant difference between OFM group and normal group in terms of serum levels of ALT (*p* = 0.1155), AST (*p* = 0.7133), ALP (*p* = 0.9966), TBIL (*p* = 0.5949), and ALB (*p* = 0.1229), which indicated an approximate return of these liver function biomarkers to normal level.

**Fig. 5:**
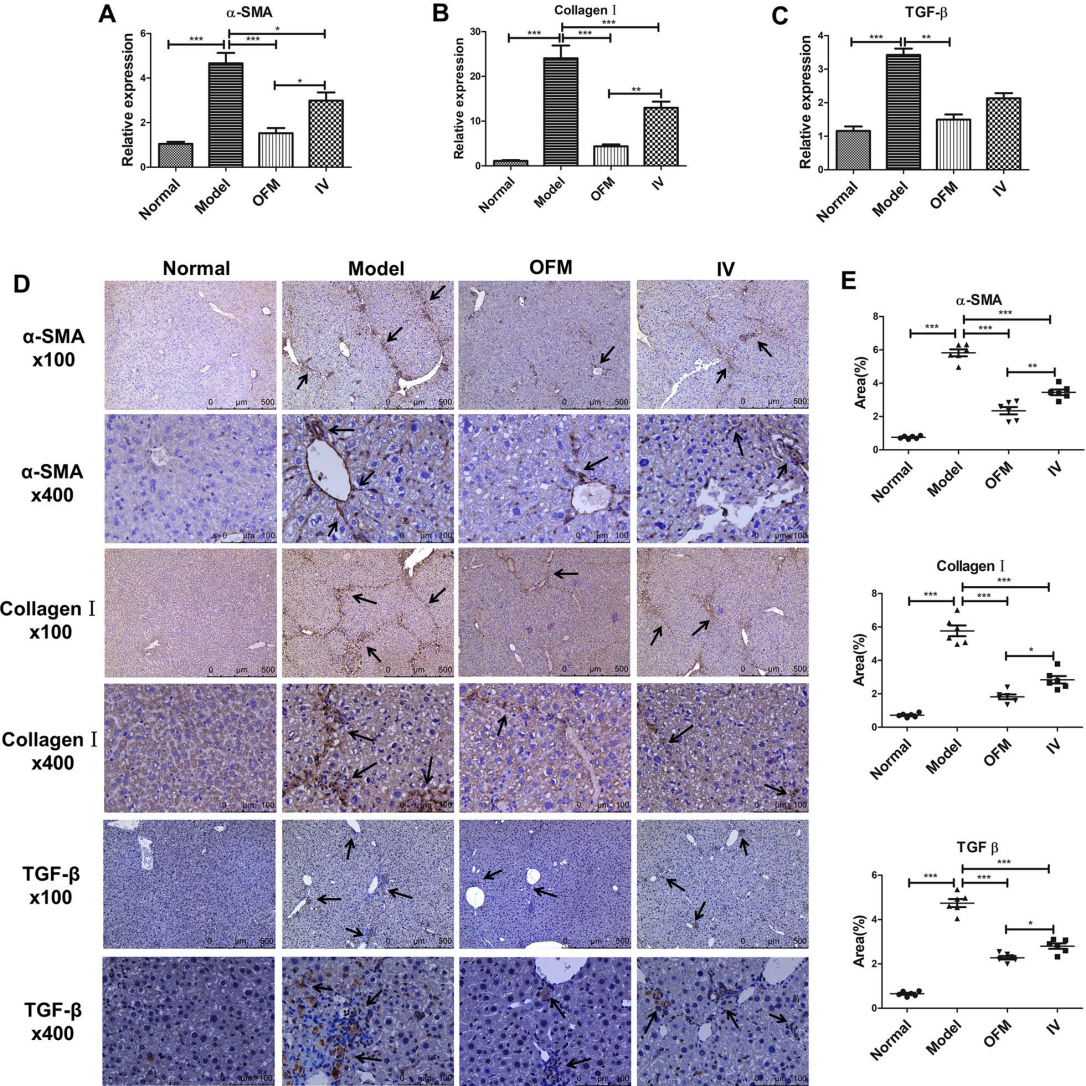
Alterations in fibrotic marker expressions after hUC-MSCs transplantion. **A–C** Relative expression levels of α-SMA, Collagen I and TGF-β mRNA in four groups. **D** IHC staining showed alterations in protein levels of α-SMA, collagen I and TGF-β in four groups (magnification ×100, ×400). **E** Quantification of areas positive for α-SMA, collagen I, and TGF-β protein expressions in all groups by Image J software (*n* = 6). Data were represented as mean ± SEM (*p < 0.05, **p < 0.01, ***p < 0.001)

The mRNA expression levels of α-SMA and collagen I, which were the main ECM components of the fibrotic liver and widely used as fibrotic markers, were evaluated through qRT-PCR. The mRNA expression levels of TGF-β, which was known as a strong introducer of HSCs to produce excessive ECM components, were also assayed by qRT-PCR. As shown in Fig. 5A–C, model group showed significant elevation of α-SMA, collagen I, and TGF-β mRNA expression as compared to normal group. After hUC-MSCs transplantation via OFM or IV route, the mRNA expression levels of α-SMA and collagen I were decreased significantly. The relative expression of α-SMA mRNA and collagen I mRNA in OFM group showed significant difference with IV group, and there was no statistically significant difference between OFM group and normal group in terms of α-SMA (*p* = 0.7757) and collagen I (*p* = 0.4934) mRNA expression. The mRNA expression levels of TGF-β in OFM group were significantly lower than those in model group while TGF-β mRNA expression levels in IV group did not show a significant difference to the model group (*p* = 0.5650). The results indicated that OFM route appeared to have a more pronounced effect on α-SMA, collagen I, and TGF-β mRNA expression.

The protein levels of α-SMA, collagen I, and TGF-β were evaluated through IHC technique. As shown in Fig. 5D, consistent with mRNA expression results, CCl_4_ exposure induced an excessive accumulation of α-SMA, collagen I, and TGF-β, and hUC-MSCs transplantation via OFM or IV route alleviated this effect in different degrees. The protein expression of α-SMA, collagen I, and TGF-β in OFM group was lower than that in IV group. The results were confirmed by quantification of the areas positive for α-SMA, collagen I, and TGF β (Fig. 5E), which indicated that OFM route was more effective on ameliorating the induced up-regulation of α-SMA, collagen I, and TGF-β in liver fibrosis.

### In vivo liver metabolomic analysis

**Fig. 6:**
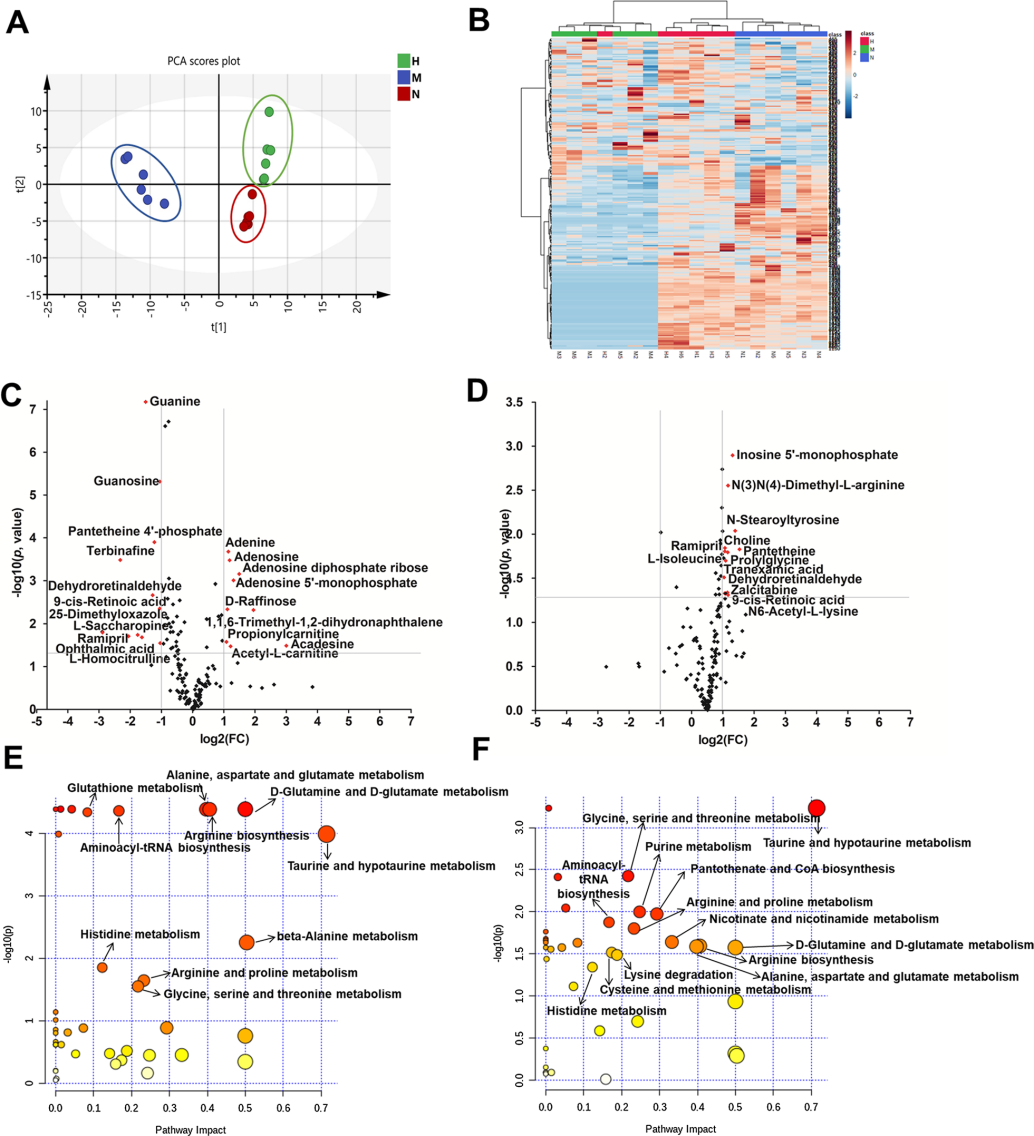
Metabolomic analysis of liver interstitial fluid via OFM sampling combining mass spectrometry detection. **A** PCA analysis of liver interstitial fluid in normal group (N), model group (M) and hUC-MSCs OFM-treated group (H) based on metabolomic analysis. **B** Heat maps of normalized metabolite concentrations in liver dialysates. Columns represent the samples, and rows represent the metabolites. The data were already normalized by using mean-centered. **C** A volcano plot based on the metabolomic data in model group *vs.* normal group. **D** A volcano plot based on the metabolomic data in hUC-MSCs OFM-treated group *vs.* model group. Metabolites with a *p* value < 0.05 and a fold change (FC) > 2.0 or < 0.5 are highlighted in the volcano plot. **E** Metabolic pathway analysis between normal group and model group. **F** Metabolic pathway analysis between hUC-MSCs OFM-treated group and model group

As an in vivo and in situ sampling technique, OFM was employed to monitor metabolic changes in liver microenvironment during progression and treatment process of hepatic fibrosis. As shown in Fig. 1B, D, OFM probe was inserted in the liver and perfused with PBS at a constant speed. Liver dialysates obtained by OFM sampling were analyzed using mass spectrometry. PCA was applied to the metabolite concentration data set (shown in Fig. 6A). The normal group (N), the model group (M) and the hUC-MSCs OFM-treated group (H) could be well separated on PCA score plots, indicating that pathological condition and treatment intervention induced metabolic perturbations in liver microenvironment. Hierarchical clustering based on metabolite patterns of liver dialysates samples was also performed and the result was presented as heat maps (Fig. 6B). It’s shown that even though model group and hUC-MSCs OFM-treated group overlapped slightly, most samples clearly grouped into three differentiated clusters, which was consistent with PCA analysis result. Individual metabolites in model group *vs.* normal group or hUC-MSCs OFM-treated group *vs.* model group were plotted in volcano plot (Fig. 6C, D). The specific metabolites that changed significantly (*p* value < 0.05 and fold change (FC) > 2.0 or < 0.5) during progression and treatment process of liver fibrosis were highlighted. As shown in Fig. 6C, it was found that the levels of 9 metabolites in liver dialysates of model group were significantly higher compared with those in the normal group, and the levels of 10 metabolites were significantly down-regulated. Figure 6D showed that 12 metabolites were significantly up-regulated in the hUC-MSCs OFM-treated group compared to the model group. It was worth noting that the levels of 9-cis-retinoic acid and dehydroretinaldehyde, which were significantly decreased in model group compared to the normal group, showed the opposite trend in hUC-MSCs OFM-treated group compared to the model group. The findings indicated a potential role of hUC-MSCs in regulating the levels of 9-cis-retinoic acid and dehydroretinaldehyde.

The metabolite concentration data set was imported into MetaboAnalyst website to explore the significantly disturbed metabolic pathways under the conditions of this study. Figure 6E, F showed the overview of metabolic pathway analysis, in which pathway with impact factor > 0.10 and *p* value < 0.05 was considered as the potential target pathway. It was shown that 10 metabolic pathways were selected as the potential pathways associated with the development of liver fibrosis, 8 of which were regulated by treatment with hUC-MSCs, including alanine, aspartate and glutamate metabolism, D-glutamine and D-glutamate metabolism, arginine biosynthesis, aminoacyl-tRNA biosynthesis, taurine and hypotaurine metabolism, histidine metabolism, arginine and proline metabolism, and glycine, serine and threonine metabolism.

## Discussion

The aims of this study were to improve hUC-MSCs administration routes in liver fibrosis models by developing a minimally-invasive OFM, which was simultaneously employed as an in vivo and in situ sampling technique to monitor liver metabolic microenvironment during fibrosis progression and healing of liver fibrosis. To evaluate the therapeutic potential of OFM transplantation of hUC-MSCs in liver fibrosis, OFM route was compared with IV route in terms of hUC-MSCs engraftment at the fibrotic liver, liver histopathological features, liver function and fibrotic markers expression after hUC-MSCs administration. Metabolomic analysis of liver dialysates obtained by OFM sampling was performed to investigate metabolic changes in liver microenvironment.Our results showed that compared with IV, OFM route caused more hUC-MSCs accumulation in the liver. Histopathological data revealed that OFM route was more effective in reducing collagen deposition in fibrotic liver and improving liver structure than IV route. OFM transplantation of hUC-MSCs significantly reduced blood ALT, AST, ALP and TBIL levels and increased ALB levels, to a greater extent than IV route. Consistent with the histopathological and biochemical results, OFM route appeared to have a more pronounced effect on ameliorating the CCl_4_-induced upregulation of the fibrotic markers, such as α-SMA, collagen I and TGF-β. Metabolomic analysis of liver dialysates showed that pathological condition and treatment intervention induced metabolic perturbations in liver microenvironment. Specially, the levels of 9-cis-retinoic acid and dehydroretinaldehyde, which were significantly decreased in model group compared to the normal group, showed opposite trend in hUC-MSCs OFM-treated group compared to the model group. Moreover, 8 metabolic pathways, which were most likely to be associated with the liver fibrosis progression, were regulated by treatment with hUC-MSCs. In summary, our study strongly suggested the high therapeutic potential of utilizing OFM route in the administration of hUC-MSCs for treatment of liver fibrosis and monitoring of the liver metabolic microenvironment in vivo.

Considering the complexity of liver fibrosis progression, efficient therapy options are severely limited. Cell therapy, particularly involving MSCs, has gained great interest for the treatment of liver diseases [26]. It has been confirmed that MSCs administration could inhibit CCl_4_-induced liver fibrosis in mice [27, 28]. The optimum route of administration is an important clinical issue for stem cell therapy. The accessibility of the transplanted MSCs to their expectable target tissue and the efficacy of therapy are strongly dependent on the administration route [29]. Hence, the transplantation route adopted in the stem cell therapy is supposed to be tailored to the lesion type and customized according to the mechanism of action of MSCs. IV is most common administration route adopted by current studies and the benefits of IV delivery in liver disease treatment have been demonstrated. Sun et al. reported that an obvious improvement of liver function was observed in acute liver failure (ALF) rats following transplantation of bone marrow stromal cells (BMSCs) via IV route [30]. Xuan et al. proved that IV delivery of hUC-MSCs could evidently inhibit liver fibrosis [31]. However, it has been well documented that MSCs transplanted via IV route were mainly accumulated in the lung and spleen, thereby showed a low presence in the injured liver tissue [32]. As expected, similar scenario was observed in our study: although the liver and spleen showed MSCs presence, IV route indeed caused more cell entrapment in the lung. Upon IV delivery, hUC-MSCs went into the systemic circulation directly. It’s widely reported that the lung is the first tissue where transplanted cells migrate [33–35]. Gao and Kurtz et al. suggested that the average size of MSCs was larger than the diameter of lung capillaries, so the larger MSCs may not be able to pass through the lung capillaries and accumulated in the lung, which was indirectly proved by the decreased homing of labeled MSCs to the lung in rats after the pretreatment with a vasodilator, sodium nitroprusside, prior to MSCs administration [36, 37]. The MSCs trapped in the lung were washed away by abundant blood flow in the pulmonary circulation, so MSCs could escape to other tissues. There are plenty of reported evidences that the homing potential of MSCs is influenced by chemokines and other molecules [38]. The primary factors attributed to the entrapment of MSCs within spleens were probably the inflammatory environment of spleens. Cell entrapment in the lung may be a hindrance for MSCs to fully display its therapeutic effects. In our study, the therapeutic effect of hUC-MSCs therapy with IV route was modest and transient. To increase the number of cells that reach to the injury site, increased initial cell dose is required, which leads to tremendous cell loss. Wang and others found a dose as high as 5 × 10^6^ cells/mouse to observe any effect in colitis treatment with IV transplantation of MSCs [39]. Wang also found that for IV injection, when the cell dose was increased, the mortality of mice would be elevated because of potential pulmonary cell embolus. Hoogduijn et al. reported that myocardial infarction (MI) in healthy vasculature was induced even though IV injection dose was 0.5 × 10^6^ MSCs per kg body weight, and a high dose was also more likely to induce a severe systemic immune response [40]. As a result, the IV route of administration requires several injections, and such huge demand of MSCs brings a cell source issue, which is an ongoing technical and operational challenge.

To bypass cell entrapment in the lung, alternative administration routes, such as hepatic artery injection, portal vein injection, intrahepatic injection and intraperitoneal injection have been investigated in several studies. Sang et al. found that MSCs transplantation through intraportal injection was superior to hepatic intra-arterial injection, intrahepatic injection and peripheral intravenous injection for ALF treatment in swine, which could improve liver function and prolong the life span of swine with ALF [29]. In our study, OFM was employed for orthotopic transplantation of hUC-MSCs into the fibrosis liver. OFM is widely reported as an in vivo sampling technology based on microdialysis (MD) with advantages of minor invasiveness, sampling continuity [41]. However, unlike MD probes, OFM probes are free of semi-permeable membrane, which is replaced by macroscopic holes. Therefore, direct access to the tissue milieu is achieved using OFM probes and sampling of interstitial components is not limited by molecular weight or lipophilicity. For example, OFM has been repeatedly reported as an alternative to MD in sampling of large molecules such as albumin and insulin or lipophilic drugs like ketoprofen [42–44]. Nevertheless, it has been rarely reported that OFM can be used for orthotopic administration of substances into different tissues in vivo. To our knowledge, the potential of OFM transplantation of hUC-MSCs for treatment of liver fibrosis was firstly explored in this study. Compared with IV route, OFM route allowed a greater number of transplanted cells to seed into the fibrotic liver, consequently, hUC-MSCs did not immediately enter to the blood flow and could fully display its therapeutic effects. Additionally, OFM route didn’t suffer from pulmonary embolism issue. In our study, OFM transplantation of 1 × 10^7^ cell/mouse was even tried, but no embolism-related death occurred. Combining histopathological and biochemical data and the results of fibrotic markers levels, it was suggested OFM transplantation of hUC-MSCs into livers was a promising technique for treatment of liver fibrosis. However, it’s worth noting that there are still several defects in OFM transplantation of hUC-MSCs. Firstly, the operation difficulty of OFM route is higher compared to IV route. Secondly, as the OFM probe is thin, the injection rate of hUC-MSCs suspension should be limited to avoid probe clogging. Thirdly, although the OFM technique is minimally invasive, the risk of liver bleeding still exists.

It is a great challenge to completely understand the action mechanisms of liver fibrosis and hUC-MSCs treatment for the disease. Metabolomics is a powerful tool to reveal the potential targets for therapeutic intervention by identifying significantly altered metabolites relevant to the progression of the disease and the healing process [45]. However, metabolomics strategy is commonly performed with collected tissues as subjects, which may give rise to contamination of samples and bias of the metabolite concentrations, resulting in misinterpretation of the results [22, 46]. Hence, in our study, the simultaneously in vivo and in situ sampling of liver interstitial fluid via OFM, and subsequently combined with metabolomic analysis, will contribute to obtain solid information on metabolome alterations. To evaluate the whether OFM sampling affects the primitive metabolomics of liver tissues, 12 mice were divided into OFM group and control group. Mice in OFM group were carried out OFM sampling of liver interstitial fluid and mice in control group were not given any intervention. Then the livers in two groups were harvested for metabolomic analysis. PCA was applied to the metabolite concentration data set. As shown in Additional file 1: Fig. 2, OFM group and control group could not be separated on PCA score plots, which demonstrated that OFM sampling had little effect on the primitive metabolomics of liver tissues. Metabolomic analysis of liver interstitial fluid showed that the levels of 9-cis-retinoic acid and dehydroretinaldehyde, which were significantly increased with the development of CCl_4_-induced hepatic fibrosis, were reversed by transplantation of hUC-MSCs. 9-cis-retinoic acid and dehydroretinaldehyde belongs to the class of organic compounds known as retinoids, and 9-cis-retinoic acid is a ligand of retinoic acid X receptor (RXR). Previous study found that the 9-cis-retinoic acid and synthetic RXR agonists had an inhibition effect on synthesis of fibronectin and collagen I, and HSC proliferation [47]. Lee reported that 9-cis-retinoic acid treatment reduced the TGF-β1 mRNA levels in L929 fibroblasts [48]. Further study revealed that the activation of PPAR gamma-RXR heterodimer repressed the TGF-β1 gene. Therefore, 9-cis-retinoic acid and dehydroretinaldehyde possibly were the potential hUC-MSCs-targeted metabolites in treatment of liver fibrosis.

Using metabolic pathway analysis, we identified 8 metabolic pathways, including alanine, aspartate and glutamate metabolism, D-glutamine and D-glutamate metabolism, arginine biosynthesis, aminoacyl-tRNA biosynthesis, taurine and hypotaurine metabolism, histidine metabolism, arginine and proline metabolism, and glycine, serine and threonine metabolism, which were significantly regulated in hUC-MSCs-treated group versus the model group. These metabolic pathways may play a crucial role in underlying therapeutical mechanism of hUC-MSCs in liver fibrosis. Du et al. recently reported that the transdifferentiation of HSCs to myofibroblastic (MF)-HSCs was strongly dependent on glutamine [49]. The α-ketoglutarate, as the end-product of D-glutamine and D-glutamate metabolism, helped to replenish the TCA cycle to satisfy the biosynthetic demands of highly proliferative MF-HSCs [50]. Glutamine metabolism was mediated by liver fibrosis related signal transduction pathway, such as TGF-β, hedgehog (Hh), hypoxia-inducible factor 1α (HIF-1α) and wnt signaling pathways[51]. Arginine metabolism generates nitric oxide (NO) by NO synthase. It’s reported that in acute liver injury, NO had the ability to oxidase lipids, proteins and DNA, promoting cell injury and death [52]. It was found that the activation of arginase located in arginine biosynthesis pathway accelerated the resolution of inflammation and promoted tissue repair [53, 54]. Taurine played vital roles in suppressing reactive oxygen species (ROS) formation and restoring mitochondrial function. Taurine administration to rats with liver fibrosis protected the liver from oxidative damage [55]. Nevertheless, Wu et al. observed that levels of taurine were elevated in CCl_4_-induced chronic liver injury [56]. The over activation of the conversion from cysteine to taurine may result in GSH depletion, which in turn, exacerbated liver damage. Shi et al. reported that MSCs significantly altered aminoacyl-tRNA biosynthesis of liver-resident immune cells in acute liver injury model [27]. Gloria et al. found that alterations in blood metabolites participating in glycine, serine and threonine metabolism coexisted with active profibrotic transcriptomic programs such as TGF-β signaling pathways, ECM-receptor interaction and cell adhesion molecules pathway [57]. Even though the roles of these key metabolic pathways needed further validation in our study, our results gave new insights into the mechanisms of MSC-based liver fibrosis therapy.

The results presented in this study showed the benefits of using a topical transplantation technique over systemic route. This effective route could possibly be further investigated as an alternative delivery route via B ultrasound or CT-guided percutaneous puncture in clinical therapy and may prove particularly valuable if the liver is the main target organ involved, or if the patient is unable to tolerate systemic exposure of MSCs. Furthermore, OFM systems could be extended to direct local delivery of chemical drugs to the liver in clinical experiments. In addition, the advantages of combining OFM sampling of liver interstitial fluid for microenvironment monitoring have been highlighted in this study. Enabling in vivo detection of minor metabolic changes, OFM could serve as a monitoring tool in clinical routine beyond scientific investigations. Nevertheless, it’s worth noting that there are still a few limitations in the study. The comparison between OFM route and other common local delivery routes such as intraportal injection and intrahepatic injection in terms of stem cell entrapment in liver and therapeutic effect is not performed. Furthermore, as it is widely believed that the liver repair effect of MSCs mainly depend on their paracrine manner by which MSCs secrete various soluble factors, the monitoring of cytokines in liver environment can be included in addition to small molecular metabolites by OFM sampling, which would promote mechanism studies of stem cell therapy.

## Conclusion

In summary, a minimally invasive OFM technique, which could be used not only to deliver hUC-MSCs to the liver but also to sample liver interstitial fluid in vivo, was successfully developed in this study. It was demonstrated that OFM transplantation of hUC-MSCs was superior to IV route in treatment of liver fibrosis due to its advantages of promoting hUC-MSCs delivery to the target tissue, inhibiting collagen deposition, improving liver function and reducing expression of fibrotic markers. In vivo monitoring of liver microenvironment demonstrated the metabolic perturbations induced by pathological condition and treatment intervention. Following metabolomic analysis of liver dialysates revealed that hUC-MSCs treatment reversed the levels of 9-cis-retinoic acid and dehydroretinaldehyde that were significantly increased with the development of CCl_4_-induced hepatic fibrosis. 8 metabolic pathways were significantly regulated after hUC-MSCs treatment in liver microenvironment, including alanine, aspartate and glutamate metabolism, D-glutamine and D-glutamate metabolism, arginine biosynthesis, aminoacyl-tRNA biosynthesis, taurine and hypotaurine metabolism, histidine metabolism, arginine and proline metabolism, and glycine, serine and threonine metabolism. Our results indicated that OFM transplantation of hUC-MSCs and OFM sampling of interstitial fluid could possibly be applied in clinical therapy and therapeutic mechanisms study in the future.

## Supplementary Information

**Additional file 1**. **Supplementary Figure 1.** The characteritics of hUC-MSCs. **A** Morphology of hUC-MSCs at passage 4. **B** Surface markers of the isolated and cultured hUC-MSCs were detected using flow cytometry. More than 95% hUC-MSCs expressed CD73, CD90, CD105, and CD29, but not CD34, CD45, CD117, and HLA-DR. **C** Fluorescence image of CM-Dil-labeled hUC-MSCs. **Supplementary Figure 2.** PCA analysis of livers obtained from control and OFM groups based on metabolomic analysis.

## Data Availability

The datasets used and/or analysed during the current study are available from the corresponding author on reasonable request.

## Abbreviations

HSCs: Hepatic stellate cells
TGF-β: Transforming growth factor beta
α-SMA: Alpha-smooth muscle actin
ECM: Extracellular matrix
HCC: Hepatocellular carcinoma
MSC: Mesenchymal stromal cell
hUC: Human umbilical cord-derived
IV: Intravenous
OFM: Open-flow microperfusion
CM-Dil: Chloromethylbenzamido-1,1′-dioctadecyl-3,3,3′,3′-tetramethyl indocarbocyanine perchlorate
PFA: Paraformaldehyde
HLA-DR: Human leukocyte antigen-DR
ALT: Aminotransferase
AST: Aspartate aminotransferase
ALP: Alkaline phosphatase
TBIL: Total bilirubin
ALB: Serum albumin
H&E: Hematoxylin&eosin
IHC: Immunohistochemistry
UPLC-ESI HR MS/MS: Ultra performance liquid chromatography-electrospray high resolution MS/MS
IS: Internal standard
HCD: High energy collisional dissociation
PCA: Principal component analysis
SEM: Standard error of the mean
ANOVA: Analysis of variance
MI: Myocardial infarction
MD: Microdialysis
MF: Myofibroblastic
Hh: Hedgehog
HIF-1α: Hypoxia-inducible factor 1α
NO: Nitric oxide
ROS: Reactive oxygen species

## References

[1] Asrani SK, Devarbhavi H, Eaton J (2019). Burden of liver diseases in the world. J Hepatol.

[2] Poelstra K (2016). Crucial steps towards any effective treatment. Nat Rev Gastroenterol Hepatol.

[3] Seki E, Schwabe RF (2015). Hepatic inflammation and fibrosis: functional links and key pathways. Hepatology.

[4] Ghavami S, Cunnington RH, Gupta S (2015). Autophagy is a regulator of TGF-beta(1)-induced fibrogenesis in primary human atrial myofibroblasts. Cell Death Dis.

[5] Higashi T, Friedman SL, Hoshida Y (2017). Hepatic stellate cells as key target in liver fibrosis. Adv Drug Deliv Rev.

[6] Ma JJ, Qiu YZ, Wang M (2019). Locostatin alleviates liver fibrosis induced by carbon tetrachloride in mice. Dig Dis Sci.

[7] Lee YA, Wallace MC, Friedman SL (2015). Pathobiology of liver fibrosis: a translational success story. Gut.

[8] Dutkowski P, Linecker M, DeOliveira ML (2015). Challenges to liver transplantation and strategies to improve outcomes. Gastroenterology.

[9] McGlynn KA, Petrick JL, London WT (2015). Global epidemiology of hepatocellular carcinoma an Emphasis on demographic and regional variability. Clin Liver Dis.

[10] Zhang D (2017). A clinical study of bone mesenchymal stem cells for the treatment of hepatic fibrosis induced by hepatolenticular degeneration. Genet Mol Res.

[11] Liu WH, Song FQ, Ren LN (2015). The multiple functional roles of mesenchymal stem cells in participating in treating liver diseases. J Cell Mol Med.

[12] Zhao L, Chen SQ, Shi XW (2018). A pooled analysis of mesenchymal stem cell-based therapy for liver disease. Stem Cell Res Ther.

[13] Wu KH, Liu YL, Zhou B (2006). Cellular therapy and myocardial tissue engineering: the role of adult stem and progenitor cells. Eur J Cardiothorac Surg.

[14] Jo CH, Kim OS, Park EY (2008). Fetal mesenchymal stem cells derived from human umbilical cord sustain primitive characteristics during extensive expansion. Cell Tissue Res.

[15] Zhang LT, Peng XB, Fang XQ (2018). Human umbilical cord mesenchymal stem cells inhibit proliferation of hepatic stellate cells in vitro. Int J Mol Med.

[16] Li ZR, Hu XJ, Mao JJ (2015). Optimization of mesenchymal stem cells (MSCs) delivery dose and route in mice with acute liver injury by bioluminescence imaging. Mol Imaging Biol.

[17] Giri J, Galipeau J (2020). Mesenchymal stromal cell therapeutic potency is dependent upon viability, route of delivery, and immune match. Blood Adv.

[18] Spriet M, Hunt GB, Walker NJ (2015). Scintigraphic tracking of mesenchymal stem cells after portal, systemic intravenous and splenic administration in healthy beagle dogs. Vet Radiol Ultrasound.

[19] Liu ZW, Mikrani R, Zubair HM (2020). Systemic and local delivery of mesenchymal stem cells for heart renovation: challenges and innovations. Eur J Pharmacol.

[20] Yin F, Wang WY, Jiang WH (2019). Human umbilical cord mesenchymal stem cells ameliorate liver fibrosis in vitro and in vivo: from biological characteristics to therapeutic mechanisms. World J Stem Cells.

[21] Hadrévi J, Ghafouri B, Sjörs A (2013). Comparative metabolomics of muscle interstitium fluid in human trapezius myalgia: an in vivo microdialysis study. Eur J Appl Physiol.

[22] Wang L, Pi Z, Liu S (2017). Targeted metabolome profiling by dual-probe microdialysis sampling and treatment using Gardenia jasminoides for rats with type 2 diabetes. Sci Rep.

[23] Chen L, Zhang C, Chen L (2017). Human menstrual blood-derived stem cells ameliorate liver fibrosis in mice by targeting hepatic stellate cells via paracrine mediators. Stem Cells Transl Med.

[24] Li N, Li SM, Li T (2020). Co-Incorporated mesoporous carbon material-assisted laser desorption/ionization ion source as an online interface of in vivo microdialysis coupled with mass spectrometry. Anal Chem.

[25] Li T, Yang H, Li X (2021). Open-flow microperfusion combined with mass spectrometry for in vivo liver lipidomic analysis. Analyst.

[26] Ng NN, Thakor AS (2020). Locoregional delivery of stem cell-based therapies. Sci Transl Med.

[27] Shi XW, Liu JQ, Chen DY (2019). MSC-triggered metabolomic alterations in liver-resident immune cells isolated from CCl4-induced mouse ALI model. Exp Cell Res.

[28] Mazhari S, Gitiara A, Baghaei K (2020). Therapeutic potential of bone marrow-derived mesenchymal stem cells and imatinib in a rat model of liver fibrosis. Eur J Pharmacol.

[29] Sang JF, Shi XL, Han B (2016). Intraportal mesenchymal stem cell transplantation prevents acute liver failure through promoting cell proliferation and inhibiting apoptosis. Hepatobiliary Pancreatic Dis Int.

[30] Sun L, Fan X, Zhang L (2014). Bone mesenchymal stem cell transplantation via four routes for the treatment of acute liver failure in rats. Int J Mol Med.

[31] Xuan J, Feng W, An ZT (2017). Anti-TGFβ-1 receptor inhibitor mediates the efficacy of the human umbilical cord mesenchymal stem cells against liver fibrosis through TGFβ-1/Smad pathway. Mol Cell Biochem.

[32] Gholamrezanezhad A, Mirpour S, Bagheri M (2011). In vivo tracking of In-111-oxine labeled mesenchymal stem cells following infusion in patients with advanced cirrhosis. Nucl Med Biol.

[33] Liu L, He H, Liu A (2015). Therapeutic effects of bone marrow-derived mesenchymal stem cells in models of pulmonary and extrapulmonary acute lung injury. Cell Transplant.

[34] Wagner B, Henschler R (2013). Fate of intravenously injected mesenchymal stem cells and significance for clinical application. Adv Biochem Eng Biotechnol.

[35] Kraitchman DL, Tatsumi M, Gilson WD (2005). Dynamic imaging of allogeneic mesenchymal stem cells trafficking to myocardial infarction. Circulation.

[36] Gao J, Dennis JE, Muzic RF (2001). The dynamic in vivo distribution of bone marrow-derived mesenchymal stem cells after infusion. Cells Tissues Organs.

[37] Kurtz A (2008). Mesenchymal stem cell delivery routes and fate. Int J Stem Cells.

[38] Honczarenko M, Le Y, Swierkowski M (2006). Human bone marrow stromal cells express a distinct set of biologically functional chemokine receptors. Stem cells.

[39] Wang M, Liang C, Hu H (2016). Intraperitoneal injection (IP), intravenous injection (IV) or anal injection (AI)? Best way for mesenchymal stem cells transplantation for colitis. Sci Rep.

[40] Hoogduijn MJ, Roemeling-van Rhijn M, Engela AU (2013). Mesenchymal stem cells induce an inflammatory response after intravenous infusion. Stem Cells Dev.

[41] Jadhav SB, Khaowroongrueng V, Derendorf H (2016). Microdialysis of large molecules. J Pharm Sci.

[42] Bodenlenz M, Ellmerer M, Schaupp L (2015). Bioavailability of insulin detemir and human insulin at the level of peripheral interstitial fluid in humans, assessed by open-flow microperfusion. Diabetes Obes Metab.

[43] Pickl KE, Magnes C, Bodenlenz M (2007). Rapid online-SPE-MS/MS method for ketoprofen determination in dermal interstitial fluid samples from rats obtained by microdialysis or open-flow microperfusion. J Chromatogr B.

[44] Ellmerer M, Schaupp L, Brunner GA (2000). Measurement of interstitial albumin in human skeletal muscle and adipose tissue by open-flow microperfusion. Am J Physiol Endocrinol Metab.

[45] Tan G, Zhou Q, Liu K (2018). Cross-platform metabolic profiling deciphering the potential targets of Shenfu injection against acute viral myocarditis in mice. J Pharm Biomed Anal.

[46] Scalbert A, Brennan L, Fiehn O (2009). Mass-spectrometry-based metabolomics: limitations and recommendations for future progress with particular focus on nutrition research. Metabolomics.

[47] Hellemams K, Verbuyst P, Quartier E (2004). Differential modulation of rat hepatic stellate phenotype by natural and synthetic retinoids. Hepatology.

[48] Lee SJ, Yang EK, Kim SG (2006). Peroxisome proliferator-activated receptor-gamma and retinoic acid X receptor alpha represses the TGF beta 1 gene via PTEN-mediated p70 ribosomal S6 kinase-1 inhibition: role for Zf9 dephosphorylation. Mol Pharmacol.

[49] Du K, Hyun J, Premont RT (2018). Hedgehog-YAP signaling pathway regulates glutaminolysis to control activation of hepatic stellate cells. Gastroenterology.

[50] Hou W, Syn WK (2018). Role of metabolism in hepatic stellate cell activation and fibrogenesis. Front Cell Dev Biol.

[51] Khomich O, Ivanov AV, Bartosch B (2020). Metabolic hallmarks of hepatic stellate cells in liver fibrosis. Cells.

[52] Ganz T, Wainstein J, Gilad S (2017). Serum asymmetric dimethylarginine and arginine levels predict microvascular and macrovascular complications in type 2 diabetes mellitus. Diabetes Metab Res Rev.

[53] Martinez FO, Helming L, Gordon S (2009). Alternative activation of macrophages: an immunologic functional perspective. Annu Rev Immunol.

[54] Bogdan C (2001). Nitric oxide and the immune response. Nat Immunol.

[55] Devi SL, Anuradha CV (2010). Mitochondrial damage, cytotoxicity and apoptosis in iron-potentiated alcoholic liver fibrosis: amelioration by taurine. Amino Acids.

[56] Wu F, Zheng H, Yang ZT (2017). Urinary metabonomics study of the hepatoprotective effects of total alkaloids from Corydalis saxicola Bunting on carbon tetrachloride-induced chronic hepatotoxicity in rats using H-1 NMR analysis. J Pharm Biomed Anal.

[57] Sanchez-Antolín G, Almohalla-Alvarez C, Bueno P (2015). Evidence of active pro-fibrotic response in blood of patients with cirrhosis. PLoS ONE.

