# Supplementary material for: A novel minimally invasive OFM technique with orthotopic transplantation of hUC-MSCs and in vivo monitoring of liver metabolic microenvironment in liver fibrosis treatment
Source: Stem Cell Res Ther. 2021 Oct 9;12:534. doi: 10.1186/s13287-021-02599-w (PMC8502355; doi:10.1186/s13287-021-02599-w)
Supplement: Supplementary file 1 — Additional file 1. Supplementary Figure 1. The characteritics of hUC-MSCs. A Morphology of hUC-MSCs at passage 4. B Surface markers of the isolated and cultured hUC-MSCs were detected using flow cytometry. More than 95% hUC-MSCs expressed CD73, CD90, CD105, and CD29, but not CD34, CD45, CD117, and HLA-DR. C Fluorescence image of CM-Dil-labeled hUC-MSCs. Supplementary Figure 2. PCA analysis of livers obtained from control and OFM groups based on metabolomic analysis. [file 13287_2021_2599_MOESM1_ESM.docx]

**A Novel Minimally Invasive OFM Technique with Orthotopic Transplantation of hUC-MSCs and *in vivo* Monitoring of Liver Metabolic Microenvironment in Liver Fibrosis Treatment**

Hui Yang^1^, Yuanyuan Xie^1^, Tuo Li^2^, Shuo Liu^1^, Sheng Zeng^1^, Bin Wang^*,1^

**^1^**Center for Clinic Stem Cell Research, the Affiliated Drum Tower Hospital of Nanjing University Medical School, Nanjing, Jiangsu, 210008, China

^2^Department of Nuclear Medicine, Peking Union Medical College Hospital, Beijing, 100730, China.

^*^ **Correspondence:** Bin Wang, Ph.D., 321 Zhongshan Road, Nanjing 210008, China. Telephone: 86-25-68182508; e-mail: [wangbin022800@126.com](mailto:wangbin022800@126.com).


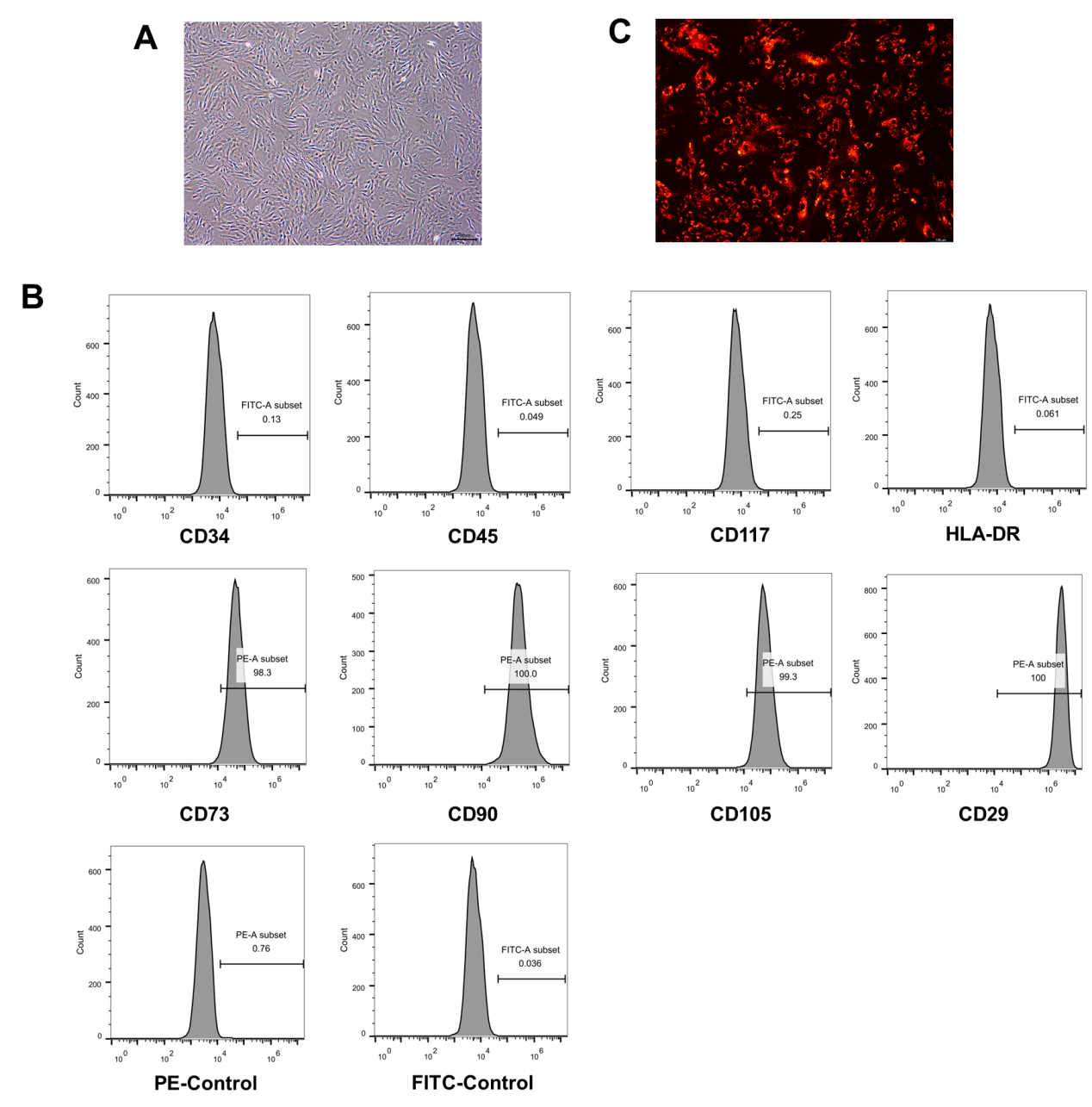


**Supplementary Figure 1.** The characteritics of hUC-MSCs. **A.** Morphology of hUC-MSCs at passage 4. **B.** Surface markers of the isolated and cultured hUC-MSCs were detected using ﬂow cytometry. More than 95% hUC-MSCs expressed CD73, CD90, CD105, and CD29, but not CD34, CD45, CD117, and HLA-DR. **C.** Fluorescence image of CM-Dil-labeled hUC-MSCs.


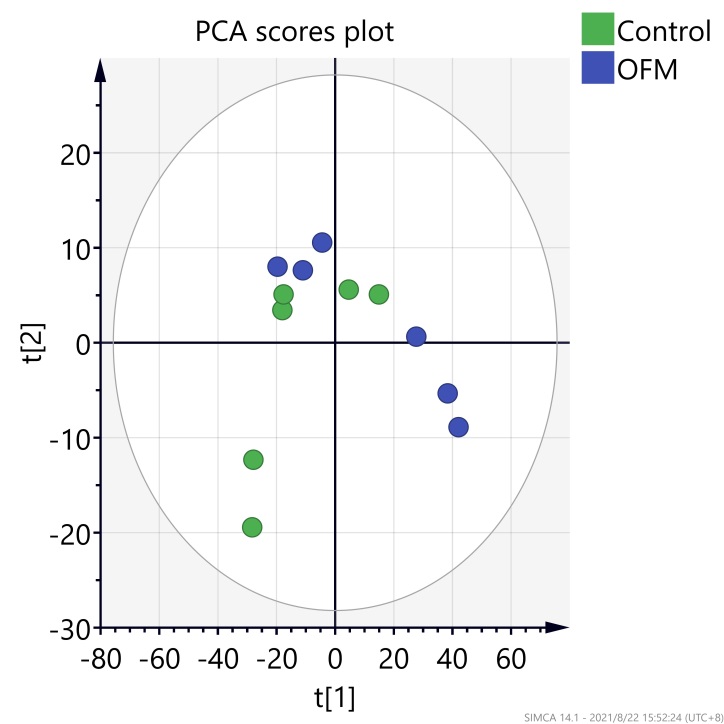


**Supplementary Figure 2.** PCA analysis of livers obtained from control and OFM groups based on metabolomic analysis.
